# Factors Associated with Axial Spondyloarthritis Remission in a Cohort of Saudi Patients with Longstanding Disease: A Multicenter Prospective Cohort Study

**DOI:** 10.2174/0115733971326045241016070431

**Published:** 2024-10-21

**Authors:** Abdulrahman Y. Almansouri, Eman Alsindi, Ibraheem Almani, Mohmed Basalama, Suzan Attar, Sultana Abdulaziz

**Affiliations:** 1 Department of Medicine, Division of Rheumatology, King Fahad Hospital, Jeddah, Saudi Arabia;; 2 Department of Medicine, King Faisal Specialist Hospital and Research Centre, Madinah, Saudi Arabia;; 3 Department of Medicine, Division of Rheumatology, King Abdulaziz University Hospital, Jeddah, Saudi Arabia;; 4 Department of Medicine, King Faisal Specialist Hospital and Research Centre, Jeddah, Saudi Arabia

**Keywords:** Axial spondyloarthritis, biological therapy, disease activity index, prognosis, remission, treat-to-target

## Abstract

**Background/Aim:**

Earlier treatment in axial spondyloarthritis (axSpA) was proposed to alter disease prognosis in this often-challenging condition. We aimed to assess the proportion of patients and prognostic factors associated with axSpA remission.

**Objective:**

The aim was to determine the number of patients with Ankylosing Spondylitis Disease Activity Score with C-reactive protein (ASDAS-CRP) of <2.1 (inactive/moderate disease activity). We also evaluated global functioning and health using the Assessment of Spondyloarthritis International Society-Health Index (ASAS-HI).

**Materials and Methods:**

Patients with axSpA who were receiving targeted synthetic/biological disease-modifying anti-rheumatic drug (ts/bDMARDs) treatments and visited the rheumatology units at two tertiary-care centers between December 2021 and December 2022 were prospectively interviewed. Data regarding patient demographics, disease features, active and previous ts/bDMARDs treatments, and disease activity scores were obtained. Patients were assessed using the ASDAS-CRP, ASDAS-erythrocyte sedimentation rate (ASDAS-ESR), Bath Ankylosing Spondylitis Disease Activity Index (BASDAI), and ASAS-HI.

**Results:**

Overall, 60 patients with axSpA were included in this study (women, n = 35); 25 (41.7%) and 36 (62.1%) achieved an ASDAS-CRP of <2.1 and an ASAS-HI of ≤5 (good health), respectively. Out of the 60 patients, 75% (n = 45) were treated with anti-tumor necrosis factor. Factors associated with achieving the target ASDAS-CRP included age (*p* = 0.019), sex (*p* = 0.015), employment status (*p* = 0.015), education level (*p* = 0.030), and the number of previous ts/bDMARDs treatments (*p* = 0.019). Additionally, the ASDAS-CRP strongly correlated with spinal pain and moderately correlated with the ASAS-HI, BASDAI, and the number of previous ts/bDMARDs treatments.

**Conclusions:**

Remission was observed in 41.7% of patients, indicating a challenge in achieving target disease activity. However, 62.1% attained good health. Achieving remission was associated with younger age, male sex, a higher level of education, lower level of spinal pain, better global function by ASAS-HI, being employed and receiving their first treatment with ts/bDMARDs (*i.e.* non-biologic switchers) at the time of interview. Our findings may potentially improve disease prognosis with the earlier use of ts/bDMARDs in those without favorable features by implementing an early axSpA intervention strategy.

## 1 INTRODUCTION

Axial spondyloarthritis (axSpA) is a relatively uncommon disease that affects the spine and sacroiliac joint in young individuals and has a heterogeneous presentation across various populations [1]. The underlying causes and mechanisms are not well known; however, they are thought to be related to environmental and individual risk factors [2, 3]. Clinically, patients present with an inflammatory back pain [3]. Radiographically, underlying inflammation usually results in structural changes that can be detected early by magnetic resonant imaging (MRI) and later will result in more pronounced radiographic damage that is detected by conventional X-rays [1, 4]. AxSpA can be classified into radiographic or non-radiographic axSpA, depending on whether the underlying structural changes can be detected on X-rays or not [4]. Previous studies worldwide have evaluated the characteristics of patients with axSpA undergoing ts/bDMARD treatments [5-10]. The factors associated with favorable disease outcomes include early diagnosis, young age, an elevated C-reactive protein (CRP) level, and male sex [7-9]. Furthermore, women were more likely to have non-radiographic axSpA compared to men [11].

Recent recommendations were provided by the Assessment of Spondyloarthritis International Society/European Alliance of Associations for Rheumatology for managing axSpA [12]. These guidelines designate Ankylosing Spondylitis Disease Activity Score with C-reactive protein (ASDAS-CRP) for assessing patients' response to treatment. Targeted synthetic or biological disease-modifying anti-rheumatic drug (ts/bDMARD) treatment is recommended for patients with ASDAS-CRP ≥2.1 who have failed two treatment courses of NSAIDs over three months after taking a rheumatologist’s opinion [12]. The ASDAS disease activity index has an objective inflammatory marker component and has been designed to differentiate between inactive, moderate, high, or very high disease activity [13-15]. Other tools are available to assess axSpA functional and disease activity, including the Bath Ankylosing Spondylitis Functional Index (BASFI) [16-19] and Bath Ankylosing Disease Activity Index (BASDAI) [16, 18, 19], respectively. The Assessment of Spondyloarthritis International Society-Health Index (ASAS-HI) has also been used in radiographic and non-radiographic axSpA to evaluate patients’ physical, emotional, and social functions [20-24]. The ASAS-HI collates several functions and presents a single score, making it an index of interest [22].

Despite the presence of influential factors, reliable assessment methods and as it is a multifaceted disease affecting populations differently, remission criteria remain undefined [12]. We believe that studies from our Saudi Arabian population, will help enrich the current knowledge by identifying prognostic features associated with axSpA [10]. Therefore, in this study, we aim to describe western-region Saudi patients with inactive or moderate axSpA disease activity, aiming to identify key features and risk factors associated with disease inactivity by utilizing ASDAS-CRP and ASAS-HI scores. We hypothesized that achieving the target will be challenging and remission rates will be low.

## 2 METHODS

### 2.1 Study Design

This was a multicenter prospective cross-sectional cohort study.

### 2.2 Setting and Study Participants

Between December 2021 and December 2022, patients diagnosed with axSpA were prospectively interviewed by three adult rheumatology fellows during their visit to two tertiary care centers in Jeddah, Saudi Arabia. The first center was King Fahad Hospital, which is the central and largest public hospital in the city of Jeddah that has a population of 3.72 million. King Fahad Hospital in Jeddah receives referrals from secondary and primary care centers in the city. The second center was the Department of Medicine at King Abdulaziz University Hospital in Jeddah. It was founded in 1977 and is the leading University based hospital in the region. A questionnaire was used to collect data on patient demographics, smoking status (none, current or previous), presence of morning stiffness, symptom duration (from disease onset, including latency period), disease duration (from the time of initial diagnosis by a rheumatologist), and axSpA domains (including inflammatory bowel disease, uveitis, psoriatic arthritis, or peripheral arthritis and comorbid illnesses). Moreover, data on previous and active treatment with non-steroidal anti-inflammatory drugs (NSAIDs), prednisolone, conventional synthetic disease-modifying anti-rheumatic drugs (csDMARDs), and ts/bDMARDs were recorded.

### 2.3 Study Inclusion and Exclusion Criteria

Inclusion criteria: adult patients aged ≥18 years who fulfilled the radiographic arm of 2009 ASAS axSpA classification criteria [25] and were diagnosed by a rheumatologist with axSpA were included. Those who failed two treatment courses of NSAIDs over three months and had a rheumatologist’s opinion to start ts/bDMARDs were included (*i.e.*, all patients were receiving ts/bDMARD treatments) [12].

We excluded patients undergoing csDMARD monotherapy, those with peripheral arthritis alone (without an axial component), those with mimicker conditions (*i.e.*, undergoing intensive physical training, and those with chronic infection (*i.e.*, tuberculosis or brucellosis).

### 2.4 Laboratory Data and Outcome Measures

Laboratory data regarding human leukocyte antigen-B27 (HLA-B27) positivity (when available) and acute-phase reactants, the CRP level, and erythrocyte sedimentation rate (ESR) were collected. Ethylenediaminetetraacetic acid (EDTA) tubes were used to collect blood samples. HLA-B27 was detected by the Single Specific Primer-Polymerase Chain Reaction (SSP-PCR) utilizing the HLA-B27 Screen Real Time kit (BioDiagene; Palermo, Italy).

Furthermore, outcome measures, including ASDAS-CRP/ESR and BASDAI, and functional measures, such as BASFI and ASAS-HI, were obtained. The validated Arabic version of the questionnaire was used in this study [18, 21]. Based on the ASDAS-CRP/ESR level (ASDAS, 2018 update), patients were classified into four groups based on disease activity: inactive disease (<1.3), moderate disease activity (1.3-2.1), high disease activity (2.2-3.5), and very high disease activity (>3.5) [26]. This study’s primary outcome was the incidence of inactive or moderate-activity disease (*i.e.*, a target ASDAS-CRP of <2.1).

Regarding the BASDAI, a score of <4 was considered a clinical response indicating a low to high disease activity, whereas a score of ≥4 was considered an active disease, indicating a very high to extremely high disease activity [27]. BASFI is a tool designed to assess physical function and does not assess disease activity [16]. It includes 10 questions on functional and physical activities, scored from 0 to 10. The mean score is calculated as the final score, and a higher mean score indicates worse impairment [16].

Further, the ASAS-HI was assessed using a questionnaire comprising 17 questions, with a higher score suggesting poorer disease outcomes [22]. An ASAS-HI of ≤5, 6-11, and ≥12 indicates good, moderate, or poor health, respectively [28].

### 2.5 Ethics

The study protocol was approved by the local ethics review boards committees (protocol numbers: 1569 and 610-22) and was conducted following the principles of the 1964 Declaration of Helsinki and its later amendments. All participants provided informed consent. Furthermore, participants’ privacy and confidentiality were ensured, no identifiers were collected, and all paper and digital copies of data were stored in a secure location within the institute premises, which could only be accessed by the research team.

### 2.6 Statistical Analysis

Statistical analyses were performed using IBM SPSS Statistics for Windows, version 23 (IBM Corp., Armonk, NY, USA), and the data were visually presented using GraphPad Prism version 8 (GraphPad Software, Inc., San Diego, CA, USA). The baseline characteristics of the study variables were defined using simple descriptive statistics. Categorical and nominal variables are presented as counts and percentages, whereas continuous variables are expressed as means ± standard deviations. Categorical variables were analyzed using the chi-square test, and between-group comparisons were conducted using the independent t-test when normal data distribution was assumed. Welch’s t-test for two-group means was used as an alternative test when normal distribution was not assumed. Pearson’s correlation coefficient was used to analyze the relationship between the variables presented as means. Dependent study variables were defined as binary outcomes, and their significant predictors were determined based on a binary logistic regression model with backward conditional elimination, using an enter criterion and elimination of 0.05 and 0.10, respectively, and a confidence interval of 95%. Statistical significance was set at *p* < 0.05. The study size was a convenience sample.

## 3 RESULTS

### 3.1 Baseline Characteristics

A total of 60 patients were prospectively recruited, the majority of whom were women (n = 35, 58.3%), with a mean age of 42.65 ± 10 years. In total, 70% of participants were employed (n = 42). Additionally, most participants had never smoked (n = 40, 66.7%), held a bachelor’s degree or higher (n = 44, 73.3%), and were overweight or obese (n = 44, 73.3%), with a mean body mass index (BMI) of 28.96 ± 5.6 kg/m^2^ (Table **1**). However, three patients had mobility restrictions that prevented them from measuring their BMI.

Only 15% (n = 9) of the participants had comorbidities, including diabetes mellitus, hypertension, or non-alcoholic fatty liver disease. Moreover, only 20% (n = 12) had a relevant family history, including a history of psoriasis (n = 5, 8.3%) or axSpA (n = 3, 5%).

### 3.2 Disease Duration, Domains, and Treatments

The mean symptom and disease durations were 9.3 ± 6.3 and 8.24 ± 6.5 years, respectively. Most patients exhibited axial and peripheral spondyloarthritis phenotype (n = 38, 63.3%), whereas the rest had only an axial phenotype (n = 22, 36.7%). Only a few patients experienced morning stiffness (n = 12, 20%) lasting 30 min (n = 5, 8.3%), 60 min (n = 5, 8.3%), or >2 h (n = 2, 3.3%). Fig. (**1**) shows the distribution of the clinical axSpA domains.

All patients included in this study were receiving ts/bDMARDs treatments. As shown in Fig. (**2**) and Supplementary Table **S1**, most patients were taking standard dosing of anti-tumor necrosis factors (anti-TNFs) (n = 45, 75%), followed by Janus Kinase inhibitors (JAKi) (n = 6, 10%), interleukin-17 inhibitors (IL-17i) (n = 5, 8.3%) and others (n = 4, 6.6%) (*i.e.*, IL12/23 inhibitors and Abatacept in patients with co-existing psoriatic arthritis). In total, 55% (n = 33) of patients were receiving their first treatment with ts/bDMARDs (*i.e.*, non-switchers).

Furthermore, 18 patients received c/sDMARDs combined with ts/bDMARDs, and 52 received NSAIDs, of which 48% showed a good response to NSAIDs. Celecoxib was the most used NSAID (n = 42, 70%), followed by naproxen (n = 7, 11.7%) and ibuprofen (n = 4, 6.7%).

### 3.3 Characteristics of Patients who Achieved Target ASDAS-CRP Scores

In total, 25 (41.7%) and 32 (53.3%) patients had inactive-to-moderate disease activity based on ASDAS-CRP scores and achieved a clinical response based on BASDAI scores, respectively. Furthermore, 36 patients (62.1%) were in good health based on the ASAS-HI score (Table **2**).

Several factors were significantly associated with achieving the target ASDAS-CRP (<2.1) (Tables **3** and **4**), including younger age (*p* = 0.019), male sex (*p* = 0.015), employment (*p* = 0.015), higher level of education (*p* = 0.030), shorter disease duration (*p* < 0.001), being on their first ts/bDMARDs treatment (*p* = 0.019), and lower ASAS-HI (*p* = 0.010).

A BASDAI of <4 was significantly associated with employment (*p* = 0.042), higher education (*p* = 0.008), being on their first ts/bDMARDs treatment (*p* = 0.004), lower mean spinal pain scores (*p* < 0.001), shorter duration of morning stiffness (*p* = 0.019), and ASAS-HI (*p* < 0.001). A significantly higher BASFI value (5.07 ± 2.3) was associated with an ASDAS-CRP score ≥2.1 (*p* < 0.001), and a lower BASFI value (2.38 ± 1.5) was associated with an ASDAS to an ASDAS-CRP score <2.1 (Table **4**).

### 3.4 Correlations between ASDAS-CRP, ASAS-HI, and Clinical Features

The ASDAS-CRP strongly correlated with ASDAS-ESR (r = 0.871, *p* < 0.001) and moderately correlated with ASAS-HI (r = 0.635, *p* < 0.001), BASDAI (r = 0.682, *p* < 0.001), and the number of previous ts/bDMARD treatments (r = 0.511, *p* < 0.001). However, the ASDAS-CRP had a weak negative correlation with the duration of active ts/bDMARD treatment (r = -0.313, *p* = 0.015).

The ASAS-HI moderately correlated with BASDAI (r = 0.675, *p* <0.001), ASDAS-ESR (r = 0.540, *p* < 0.001), spinal pain (r = 0.507, *p* < 0.001), and the number of previous ts/bDMARD treatments (r = 0.366, *p* < 0.001). However, it had a weak negative correlation with the duration of active ts/bDMARD treatment (r = −0.260, *p* = 0.049) (Supplementary Table **S2**).

Furthermore, spinal pain negatively correlated with the duration of active ts/bDMARD treatment (r = -0.413, *p* = 0.001) and weakly associated with ESR (r = 0.272, *p* = 0.035), (Supplementary Table **S2**).

## 4 DISCUSSION

In this multicenter, prospective cohort study, we assessed the incidence of inactive/moderate disease-activity axSpA and the associated factors. The key findings of this study are as follows: First, 41.7% (n = 25) of patients achieved the target ASDAS-CRP (<2.1), and 62.1% (n = 36) were classified as having good global health function. Second, younger age, male sex, a higher level of education, lower level of spinal pain, better global function by ASAS-HI, employment, and being on their first ts/bDMARDs treatment were associated with achieving target disease activity. Therefore, older age, women, those with lower education, higher spinal pain, and worse global function by ASAS-HI and ts/bDMARDs switchers were associated with worse disease activity and not achieving target ASDAS-CRP. Finally, shorter disease duration was observed among patients who achieved target disease activity, and a higher number of patients with non-radiographic axSpA did not achieve their target, accounting for the majority of the study being women [11]; however, these were statistically insignificant. To the best of our knowledge, this is the first study from Saudi Arabia’s western region to report on the percentage of patients with axSpA who achieved a clinical response based on ASDAS-CRP scores, explore the factors associated with it, include a global functional assessment by the ASAS-HI, and elucidate its relationship with an earlier intervention concept for patients without favorable prognosis.

The reported remission rates for axSpA vary based on the definitions of remission and disease duration considered in the study [5, 7-9, 29]. For instance, Shimabuco *et al.* used a stringent definition for remission, reporting a remission rate of 39% [29]. They also identified baseline factors associated with remission similar to those identified in this study, including younger age, shorter disease duration, initial ts/bDMARD treatment (*i.e.*, non-switcher), and low scores on other functional and disease activity indices [29]. Furthermore, in a post-hoc analysis of the Quick and Simple And Reliable study, D’Angelo *et al.* reported that 54.6% of their patients achieved an ASDAS-CRP score of <2.1 [5]. In the Devenir des Spondylarthropathies Indifferérenciées Récentes cohort, Pina Vegas *et al.* reported factors associated with remission after a 5-year follow-up [7]. These factors, similar to those identified in the present study, included male sex and higher education levels [7]. However, they reported remission rates that were significantly lower than those observed in our study (25% of patients in their large cohort achieved remission) [7]. Furthermore, a study of remission-related factors in patients with axSpA from the ABILITY-3 cohort revealed that younger age and male sex were associated with axSpA remission, which was similar to the findings in our study [8]. However, smoking was not significantly associated with disease indices in our study, aligning with the results of other previous studies [4, 6].

Most patients in our study achieved good health based on the ASAS-HI category (≤5, n = 36, 62.1%). The ASAS-HI is a recently introduced functional measure that has not been extensively evaluated in many cohorts. We identified moderate correlations between the ASAS-HI and ASDAS-CRP (r = 0.635, *p* < 0.001), BASDAI (r = 0.675, *p* < 0.001), and ASDAS-ESR (r = 0.540, *p* < 0.001), consistent with the results of previous studies [20, 23, 30]. Most patients were women, middle-aged, and had a high BMI, but the prevalence of comorbidities was low in this relatively young population. All patients met the imaging arm of the 2009 ASAS classification criteria [25].

In this study, only a few patients with axSpA showed HLA-B27 positivity. This could be attributed to underreporting, as one-third of the patients were not tested for HLA-B27. However, HLA-B27 positivity could also be non-prevalent in this study population. Omair *et al.* reported that 60.4% and 25.9% of Saudi Arabian patients with radiographic and non-radiographic axSpA showed HLA-B27 positivity, respectively [31]. Similarly, Ziade *et al.* reported that 41.1% of Lebanese patients with axSpA tested positive for HLA-B27 [32]. Moreover, the low rates of HLA-B27 positivity have been reported as a limitation of HLA-B27 testing in Arab populations [33, 34] and more recently in other populations [35].

Based on the mean disease duration in this cohort, patients had relatively longstanding axSpA. However, the mean course of treatment with ts/bDMARDs was shorter than the disease duration. This result could be explained by the delayed initiation of ts/bDMARDs therapy due to delays in referral to tertiary centers for treatment initiation; such delays warrant further investigation. As mentioned earlier, all study participants were receiving ts/bDMARDs treatments at the time of participation, and active treatment with anti-TNFs was predominant. This outcome is anticipated because of the widespread availability of anti-TNFs, as these predate other ts/bDMARDs leading clinicians to have more experience with their use. In some institutions, hospital policy dictates that switching to another type of ts/bDMARDs, such as IL-17i or JAKi, should only be considered if two anti-TNF treatments fail. Only a few patients with axial PsA were on other therapies [36-38]. Furthermore, most patients were undergoing their first ts/bDMARDs treatment (n = 33, 55%), and 60% of them achieved the target ASDAS-CRP score of <2.1, whereas only 39% of patients who had already undergone one round of ts/bDMARDs treatment (*i.e.*, switchers, *p* = 0.019) met the goal. This indicates that non-switchers have a better chance of achieving the target ASDAS-CRP and, thus, superior disease outcomes. Shimabuco *et al.* also reported that non-switchers had better disease outcomes [29].

Lastly, the concept of a “window of opportunity” and early intervention in axSpa has recently been entertained [39, 40]. As in early rheumatoid arthritis [41, 42], an earlier intervention strategy in axSpA has been suggested to improve prognosis [39]. Recently, new axSpA biomarkers, such as fetuin-A, have been proposed to help prognosticate patients [35]. Utilizing axSpA poor prognostic factors highlighted in this study and the above-mentioned novel biomarkers might be of benefit, in view of the window of opportunity concept, in having better long-term outcomes for these patients and warrants further studies.

Our study has some limitations. First, the prevalence of HLA-B27 positivity was underreported, precluding from making associations. Second, this study had a small sample size; therefore, the results cannot be generalized. Third, we performed only univariate analysis; therefore, a more comprehensive multivariate analysis would be useful. However, this was a multicenter, real-world, prospective study that involved two disease activity indices (ASDAS-CRP and BASDAI) and two functional health indices (ASAS-HI and BASFI) to elaborate on a treat-to-target concept for remission achievement in Saudi patients with axSpA and highlight prognostic factors.

## CONCLUSION

In conclusion, patients with axSpA were characterized in this study using the ASDAS-CRP, and more than 40% of patients had achieved remission. Furthermore, more than 60% of patients were classified as having good health based on the ASAS-HI. Patients who were employed, younger, male, highly educated, receiving their first treatment with ts/bDMARDs (*i.e.*, non-switchers), and had lower spinal pain were more likely to achieve the target ASDAS-CRP than their counterparts. Despite new treatment options for axSpA, anti-TNF therapy remains the most administered treatment owing to its longer history of use. Our findings may help guide treatment decisions by being more proactive in the earlier administration of ts/bDMARDs in those with poor outcomes, and potentially improving remission rates by implementing an earlier intervention strategy.

## AUTHORS’ CONTRIBUTIONS

The authors confirm their contribution to the paper as follows:

A.Y.A. wrote the paper.

E.A. contributed to the data collection, interpretation and analysis.

M.B. and I.A. collected the data.

S.A. and S.A. analysed the data and contributed to interpretation and study concept or design.

All authors reviewed the results and approved the final version of the manuscript.

## Figures and Tables

**Fig. (1):**
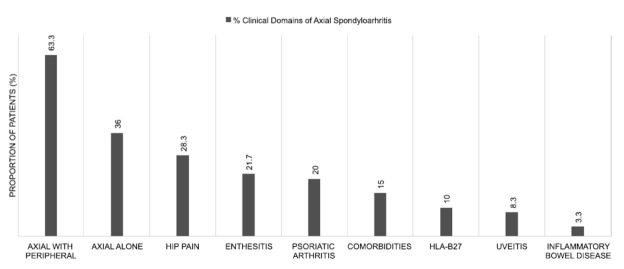
Clinical domains of axial spondyloarthritis. A higher proportion of patients had axial with peripheral arthritis compared to those with axial spondyloarthritis alone (63.3% *vs* 36%). Enthesitis and psoriatic arthritis were noted in approximately 20% of the patients. Uveitis and inflammatory bowel disease were noted in 8.3% and 3.3% of the patients, respectively.

**Fig. (2):**
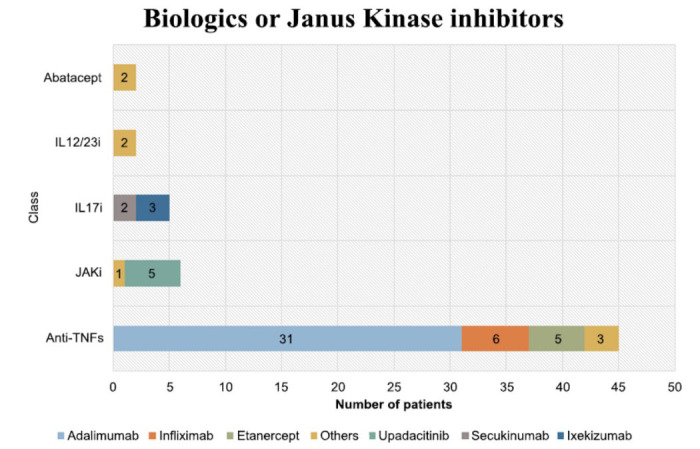
Targeted synthetic/biologic disease modifying anti rheumatic drugs received by patients. The most received targeted synthetic/biologic disease modifying antirheumatic drugs (ts/bDMARDs) were anti-tumour necrosis factors (Anti-TNFs) (75%), owing to their known efficacy, longer use in practice and being first line as per institutional preferences. The remaining 25% of patients were receiving other types of ts/bDMARDs. Limited number of switchers (6.6%) with psoriatic axial spondyloarthritis were receiving IL12/23 inhibitors or Abatacept. **Abbreviations:** Anti-TNFs, Anti-tumour necrosis factors; JAKi, Janus Kinase inhibitors; IL-17i, Interleukin 17 inhibitors (IL17i); IL12/23i, Interleukin12/23 inhibitors.

**Table 1:** Baseline characteristics, laboratory, and radiography results.

**Variable (unit)**		**n**	**Mean (± SD)**
Age (years)		60	42.65 (± 10.0)
BMI (kg/m^2^) *		57	28.96 (± 5.6)
Symptom duration (years)		60	9.30 (± 6.3)
Disease duration (years)		60	8.24 (± 6.5)
Spinal pain (0-10) ^†^		60	4.83 (± 2.3)
Duration of active ts/bDMARD treatment (years)		60	3.05 (± 2.2)
Duration of previous ts/bDMARD treatment for switchers (years)		24	2.33 (± 2.9)
CRP (mg/dL)		60	6.93 (± 8.0)
ESR (mm/h)		60	25.15 (± 25.6)
**Results**	**Category**	**n**	**%**
Total	-	60	100.0
Age	25-35 years	19	31.7
	36-45 years	16	26.7
	46-55 years	19	31.7
	>55 years	6	10.0
Sex	Male	25	41.7
	Female	35	58.3
Occupation	Unemployed	18	30.0
	Employed	42	70.0
Education	Below bachelor’s degree	16	26.7
	Bachelor’s degree and higher	44	73.3
Smoking	None	40	66.7
	Yes, at least once	20	33.3
BMI *	Normal	15	26.3
	Overweight	21	36.8
	Obese	21	36.8
	Missing	3	-
axSpA domains	Dactylitis	6	10.0
	Enthesitis	13	21.7
	Hip Pain	17	28.3
	IBD	2	3.3
	Psoriatic arthritis	12	20.0
	Uveitis	5	8.3
HLA-B27 positive	Yes	6	10.0
	No	38	63.3
	Unknown	16	26.7
axSpA types	Non-radiographic	34	56.7
	Radiographic	26	43.3

**Note:** *-Missing data are due to patients’ mobility restrictions (patients using wheelchairs). †^-^Spinal pain was assessed on a visual scale from 0 to 10 cm. **Abbreviations:** BMI, body mass index; ts/bDMARDs, targeted synthetic/biological disease-modifying anti-rheumatic drugs; CRP, C-reactive protein; ESR, erythrocyte sedimentation rate; IBD, inflammatory bowel disease; HLA-B27, human leucocyte antigen-B27; axSpA, axial spondyloarthritis; SD, standard deviation.

**Table 2:** Disease activity and global functional scores for the study population.

**Variable**		**n**	**mean (SD)**
ASDAS-CRP		60	2.50 (± 1.0)
ASDAS-ESR		59	2.57 (± 1.1)
ASAS-HI		58	4.47 (± 3.9)
BASDAI		60	4.28 (± 2.5)
BASFI		59	3.98 (± 2.4)
Total		n (60)	100%
Achieved target ASDAS-CRP (<2.1)	Inactive/moderate disease activity	25	41.7
	Did not achieve the target	35	58.3
BASDAI	Inactive disease (BASDAI <4)	32	53.3
	Active disease (BASDAI ≥4)	28	46.7
ASAS-HI	≤5 Good health	36	62.1
	6-11 Moderate health	20	34.5
	≥12 Poor health	2	3.4

**Abbreviations:** ASDAS-CRP, Ankylosing Spondylitis Disease Activity Score with C-reactive-protein; ASDAS-ESR, Ankylosing Spondylitis Disease Activity Score with erythrocyte sedimentation rate, ASAS-HI: Assessment of Spondyloarthritis International Society-Health Index; BASDAI, Bath Ankylosing Spondylitis Disease Activity Index; BASFI, Bath Ankylosing Spondylitis Functional Index; SD, standard deviation.

**Table 3:** Demographic factors associate with achieving target ASDAS-CRP and BASDAI clinical response.

**Variables**		**Total**	**BASDAI**		***p*-value**	**Achieving the Target ASDAS-CRP**		***p*-value**
			**Achieving Clinical Response**	**Active Disease**		**Achieving ASDAS-CRP <2.1**	**Not Achieving the Target**	
Achieving target ASDAS-CRP	Achieving ASDAS-CRP <2.1	25	22 (88.0%)	3 (12.0%)	<0.001^§^	-	-	-
	Not achieving the target	35	10 (28.6%)	25 (71.4%)		-	-	-
BASDAI	Achieving clinical response	32	-	-	-	22 (68.8%)	10 (31.3%)	<0.001^§^
	Active disease	28	-	-	-	3 (10.7%)	25 (89.3%)	
Age	25-35 years	19	12 (63.2%)	7 (36.8%)	0.188	10 (52.6%)	9 (47.4%)	0.019^§^
	36-45 years	16	10 (62.5%)	6 (37.5%)		10 (62.5%)	6 (37.5%)	
	46-55 years	19	9 (47.4%)	10 (52.6%)		5 (26.3%)	14 (73.7%)	
	>55 years	6	1 (16.7%)	5 (83.3%)		0 (0.0%)	6 (100.0%)	
Sex	Male	25	15 (60.0%)	10 (40.0%)	0.382	15 (60.0%)	10 (40.0%)	0.015^§^
	Female	35	17 (48.6%)	18 (51.4%)		10 (28.6%)	25 (71.4%)	
Occupation	Unemployed	18	6 (33.3%)	12 (66.7%)	0.042^§^	3 (16.7%)	15 (83.3%)	0.010^§^
	Employed	42	26 (61.9%)	16 (38.1%)		22 (52.4%)	20 (47.6%)	
Education	Below bachelor’s degree	16	4 (25.0%)	12 (75.0%)	0.008^§^	3 (18.8%)	13 (81.3%)	0.030^§^
	Bachelor’s degree and higher	44	28 (63.6%)	16 (36.4%)		22 (50.0%)	22 (50.0%)	
Smoking	None	40	21 (52.5%)	19 (47.5%)	0.855	16 (40.0%)	24 (60.0%)	0.711
	Yes, at least once	20	11 (55.0%)	9 (45.0%)		9 (45.0%)	11 (55.0%)	
BMI	Normal	15	6 (40.0%)	9 (60.0%)	0.589	6 (40.0%)	9 (60.0%)	0.640
	Overweight	21	11 (52.4%)	10 (47.6%)		10 (47.6%)	11 (52.4%)	
	Obese	21	12 (57.1%)	9 (42.9%)		7 (33.3%)	14 (66.7%)	
Number of previous ts/bDMARDs	None	33	23 (69.7%)	10 (30.3%)	0.004^§^	20 (60.6%)	13 (39.4%)	0.019^§^
	1	18	6 (33.3%)	12 (66.7%)		3 (16.7%)	15 (83.3%)	
	More than 1	9	3 (33.3%)	6 (66.7%)		2 (22.2%)	7 (77.8%)	

**Note**: §-significant using chi-square test, *p*-value <0.05. **Abbreviations:** ASDAS-CRP, Ankylosing Spondylitis Disease Activity Score with C-reactive protein; BASDAI, Bath Ankylosing Spondylitis Disease Activity Index; BMI, Body mass index; ts/bDMARD, targeted synthetic/biologic disease-modifying anti-rheumatic drugs.

**Table 4:** Clinical laboratory and radiography factors associated with achieving the target ASDAS-CRP and BASDAI clinical response.

**Variables**		**Total**	**BASDAI**		***p*-value**	**Achieving the Target ASDAS-CRP**		***p*-value**
			**Achieving Clinical Response**	**Active Disease**		**Achieving ASDAS-CRP <2.1**	**Not Achieving the Target**	
Symptom duration (year)		60	5.29 ± 0.9	7.35 ± 1.4	0.643	4.74 ± 0.9	7.17 ± 1.2	0.273
Disease duration (year)		60	4.98 ± 0.9	7.92 ± 1.5	0.849	4.30 ± 0.9	7.60 ± 1.3	0.228
Spinal pain ^‡^		60	1.62 ± 0.3	2.24 ± 0.4	<0.001 *	1.47 ± 0.3	2.24 ± 0.4	<0.001 *
BASFI		59	3.19 ± 2.0	4.85 ± 2.6	0.007 *	2.38 ± 1.5	5.07 ± 2.3	<0.001 *
Arthritis distribution	Axial Alone	22	12 (54.5%)	10 (45.5%)	0.886	12 (54.5%)	10 (45.5%)	0.124
	Axial with Peripheral	38	20 (52.6%)	18 (47.4%)		13 (34.2%)	25 (65.8%)	
Duration of morning stiffness	0 hours	48	30 (62.5%)	18 (37.5%)	0.019 **	24 (50.0%)	24 (50.0%)	0.062
	30 minutes	5	2 (40.0%)	3 (60.0%)		0 (0.0%)	5 (100.0%)	
	60 minutes	5	0 (0.0%)	5 (100.0%)		1 (20.0%)	4 (80.0%)	
	>2 hours	2	0 (0.0%)	2 (100.0%)		0 (0.0%)	2 (100.0%)	
Number of damaged joints by X-ray imaging	0	45	25 (55.6%)	20 (44.4%)	0.413	19 (42.2%)	26 (57.8%)	0.683
	2	8	3 (37.5%)	5 (62.5%)		3 (37.5%)	5 (62.5%)	
	3	1	1 (100.0%)	0 (0.0%)		0 (0.0%)	1 (100.0%)	
Family history	None	48	26 (54.2%)	22 (45.8%)	0.796	20 (41.7%)	28 (58.3%)	>0.999
	Yes	12	6 (50.0%)	6 (50.0%)		5 (41.7%)	7 (58.3%)	
axSpA	Non-radiographic	34	16 (47.1%)	18 (52.9%)	0.265	12 (35.3%)	22 (64.7%)	0.252
	Radiographic	26	16 (61.5%)	10 (38.5%)		13 (50.0%)	13 (50.0%)	
HLA-B27 positivity	Yes	6	5 (83.3%)	1 (16.7%)	0.060	3 (50.0%)	3 (50.0%)	0.871
	No	38	22 (57.9%)	16 (42.1%)		15 (39.5%)	23 (60.5%)	
	Unknown	16	5 (31.3%)	11 (68.8%)		7 (43.8%)	9 (56.3%)	
Comorbidity	Yes	9	4 (44.4%)	5 (55.6%)	0.562	2 (22.2%)	7 (77.8%)	0.199
	No	51	28 (54.9%)	23 (45.1%)		23 (45.1%)	28 (54.9%)	
ASAS-HI category	≤5 Good health	36	29 (80.6%)	7 (19.4%)	<0.001**	21 (58.3%)	15 (41.7%)	0.010 **
	6-11 Moderate health	20	3 (15.0%)	17 (85.0%)		4 (20.0%)	16 (80.0%)	
	≥12 Poor health	2	0 (0.0%)	2 (100.0%)		0 (0.0%)	2 (100.0%)	

**Note**: ‡-Spinal pain was assessed on a visual scale from 0 to 10 cm. *-significant using Welch’s t-test, *p*-value <0.05. **-significant using chi-square test, *p*-value <0.05. **Abbreviations:** ASDAS-CRP, Ankylosing Spondylitis Disease Activity Score with C-reactive protein; BASDAI, Bath Ankylosing Spondylitis Disease Activity Index; BASFI, Bath Ankylosing Spondylitis Functional Index; axSpA, axial spondyloarthritis; HLA-B27, Human leucocyte antigen-B27; ASAS-HI, Assessment of Spondyloarthritis International Society-Health Index.

## Data Availability

The data and supportive information are available within the article.

## LIST OF ABBREVIATIONS

anti-TNFs: Anti-tumor Necrosis Factors
axSpA: Axial Spondyloarthritis
BASDAI: Bath Ankylosing Spondylitis Disease Activity Index
CRP: C-reactive Protein
EDTA: Ethylenediaminetetraacetic Acid
MRI: Magnetic Resonant Imaging
NSAIDs: Non-steroidal Anti-inflammatory Drugs

## STANDARDS OF REPORTING

STROBE guidelines were followed.
