# Supplementary material for: Factors Associated with Axial Spondyloarthritis Remission in a Cohort of Saudi Patients with Longstanding Disease: A Multicenter Prospective Cohort Study
Source: Curr Rheumatol Rev. 2024 Oct 21;21(4):435–45. doi: 10.2174/0115733971326045241016070431 (PMC12606613; doi:10.2174/0115733971326045241016070431)
Supplement: Supplementary file 1 [file CRR-21-4-435_SD1.pdf]

Supplementary Material

Factors Associated with Axial Spondyloarthritis Remission in a Cohort of Saudi Patients with Longstanding Disease: A Multicenter Prospective Cohort Study

Abdulrahman Y. Almansouri[1,2], Eman Alsindi[1], Ibraheem Almani[3,4], Mohmed Basalama[1], Suzan Attar[3] and Sultana Abdulaziz[1,*]

[1]Department of Medicine, Division of Rheumatology, King Fahad Hospital, Jeddah, Saudi Arabia; [2]Department of Medicine, King Faisal Specialist Hospital and Research Centre, Madinah, Saudi Arabia; [3]Department of Medicine, Division of Rheumatology, Faculty of Medicine, King Abdulaziz University, Jeddah, Saudi Arabia; [4]Department of Medicine, King Faisal Specialist Hospital and Research Centre, Jeddah, Saudi Arabia

Supplementary Table 1. Type of axial spondyloarthritis therapy received.

**Result**		**n**	**%**
Total		60	100.0
Receiving NSAIDs	Yes	54	90
	None or others	6	10
Active ts/bDMARD treatment	Adalimumab	31	51.7
	Infliximab	6	10.0
	Etanercept	5	8.3
	Upadacitinib	5	8.3
	Certolizumab	3	5.0
	Ixekizumab	3	5.0
	Abatacept	2	3.3
	Secukinumab	2	3.3
	Ustekinumab	2	3.3
	Baricitinib	1	1.7
Active ts/bDMARD treatment class	Anti-TNFs	45	75.0
	JAKi	6	10.0
	IL-17i	5	8.3
	IL-12/23i	2	3.3
	Others	2	3.3
Number of previous ts/bDMARD	None	33	55.0
	1	18	30.0
	>1	9	15.0
csDMARDS	None	42	70.0
	Methotrexate	12	20.0
	Sulfasalazine	5	8.3
	Leflunomide	1	1.7

Abbreviations: ts/bDMARDs, targeted synthetic/biologic disease-modifying anti-rheumatic drugs; csDMARDs, conventional synthetic disease-modifying anti-rheumatic drugs; IL-12/23i, interleukin-12/23 inhibitors; IL-17i, interleukin-17 inhibitor; JAKi, Janus kinase inhibitor; anti-TNFs, anti-tumor necrosis factor drugs

1875-6360/25 **© 2025 Bentham Science Publishers**

Supplementary Table 2. Correlations between other clinical, disease activity and functional indices.

Correlating Variable		Duration of Treatment with Active ts/bDMARDs	Duration of Treatment with Previous ts/bDMARDs	Spinal Pain §	BASDAI	ASDAS-ESR	ASDAS-CRP	ASAS-HI	Disease Duration
Frequency of switching ts/bDMARDs	r	–0.287*	0.686**	0.486**	0.431**	0.451**	0.511**	0.366**	0.047
	p-value	0.026	<0.001	<0.001	0.001	<0.001	<0.001	0.005	0.722
	N	60	24	60	60	59	60	58	60
Duration of treatment with active ts/bDMARDs	r		–0.181	–0.413**	–0.401**	–0.281*	–0.313*	–0.260*	0.182
	p-value		0.397	0.001	0.001	0.031	0.015	0.049	0.164
	N		24	60	60	59	60	58	60
Duration of treatment with previous ts/bDMARDs	r			0.213	0.258	0.147	0.091	0.145	0.003
	p-value			0.318	0.223	0.493	0.674	0.518	0.987
	N			24	24	24	24	22	24
Spinal pain§	r				0.607**	0.674**	0.710**	0.507**	0.116
	p-value				<0.001	<0.001	<0.001	<0.001	0.377
	N				60	59	60	58	60
BASDAI	r					0.599**	0.682**	0.675**	–0.024
	p-value					<0.001	<0.001	<0.001	0.854
	N					59	60	58	60
ASDAS-ESR	r						0.871**	0.540**	0.156
	p-value						<0.001	<0.001	0.237
	N						59	57	59
ASDAS-CRP	r							0.635**	0.175
	p-value							<0.001	0.181
	N							58	60
ASAS-HI	r								–0.023
	p-value								0.862
	N								58

*. Correlation is significant at the 0.05 level (2-tailed).
**. Correlation is significant at the 0.01 level (2-tailed).
r, Pearson's correlation coefficient
§- Spinal pain was assessed on a visual scale from 0 to 10 cm
Abbreviations: ASDAS-CRP/ESR, Ankylosing Spondylitis Disease Activity Score with C-reactive protein/erythrocyte sedimentation rate; ASAS-HI, Assessment of Spondylarthritis International Society-Health Index; BASDAI, Bath Ankylosing Spondylitis Disease Activity Index; ts/bDMARDs, targeted synthetic/biologic disease-modifying anti-rheumatic drugs
